# Dental calculus and isotopes provide direct evidence of fish and plant consumption in Mesolithic Mediterranean

**DOI:** 10.1038/s41598-018-26045-9

**Published:** 2018-05-25

**Authors:** Emanuela Cristiani, Anita Radini, Dušan Borić, Harry K. Robson, Isabella Caricola, Marialetizia Carra, Giuseppina Mutri, Gregorio Oxilia, Andrea Zupancich, Mario Šlaus, Dario Vujević

**Affiliations:** 1 Department of Oral and Maxillo Facial Sciences, “Sapienza” University of Rome, Via Caserta 6, 00161 Rome, Italy; 2 BioArCh, Department of Archaeology, University of York, York, YO10 5YW United Kingdom; 3 The Italian Academy for Advanced Studies in America, Columbia University, 1161 Amsterdam Avenue, New York, NY 10027 USA; 4 Anthropological Center, Croatian Academy of Sciences and Arts, 10000 Zagreb, Croatia; 5 Department of Archaeology, University of Zadar, Zadar, Croatia

## Abstract

In this contribution we dismantle the perceived role of marine resources and plant foods in the subsistence economy of Holocene foragers of the Central Mediterranean using a combination of dental calculus and stable isotope analyses. The discovery of fish scales and flesh fragments, starch granules and other plant and animal micro-debris in the dental calculus of a Mesolithic forager dated to the end of the 8th millenium BC and buried in the Vlakno Cave on Dugi Otok Island in the Croatian Archipelago demonstrates that marine resources were regularly consumed by the individual together with a variety of plant foods. Since previous stable isotope data in the Eastern Adriatic and the Mediterranean region emphasises that terrestrial-based resources contributed mainly to Mesolithic diets in the Mediterranean Basin, our results provide an alternative view of the dietary habits of Mesolithic foragers in the Mediterranean region based on a combination of novel methodologies and data.

## Introduction

The Central Mediterranean has yielded a unique funerary record whereby large and small islands in the Tyrrhenian, Ionian and Adriatic Seas were selected as burial locations by Mesolithic foragers. Mesolithic burials are known from Sicily, the Egadi Islands, Sardinia, Corsica as well as some islands of the Croatian archipelago^1–9^. Despite the importance of marine localities for disposal of the dead, the dietary stable isotopic and zooarchaeological data for these Central Mediterranean foragers has emphasised that marine resources (and plant foods) had a marginal role to their diets^10–17^. This pattern is in stark contrast to Mesolithic forgers inhabiting regions along the Atlantic coastline^18,19^.

Recent methodological developments in the analysis of micro-fossils trapped in human dental calculus has provided a new way for assessing neglected aspects of hunter-gatherer-fisher subsistence along with non-dietary information on human interaction with varied environments^20,21^. The potential of this method has mainly been recognised for reconstructing the relative proportion of plant foods in human diets. It has emerged that the harvesting and processing of starchy resources, such as grasses, tubers or roots rich in carbohydrates, might not have been a sole prerogative of agricultural societies^22^. Yet, it is more difficult to assess a direct correlation between the presence of plant remains in calculus and estimations of the quantity of the plant foods consumed^23^ while micro-debris of animal origin has rarely been recovered in ancient plaque. In addition, the recovery in dental calculus of micro-particles of materials deliberately or accidentally ingested during the performance of various activities has also proven the potential of the study of dental calculus to provide insights into aspects of individual life-ways other than nutrition^20,21,24^.

**Figure 1:**
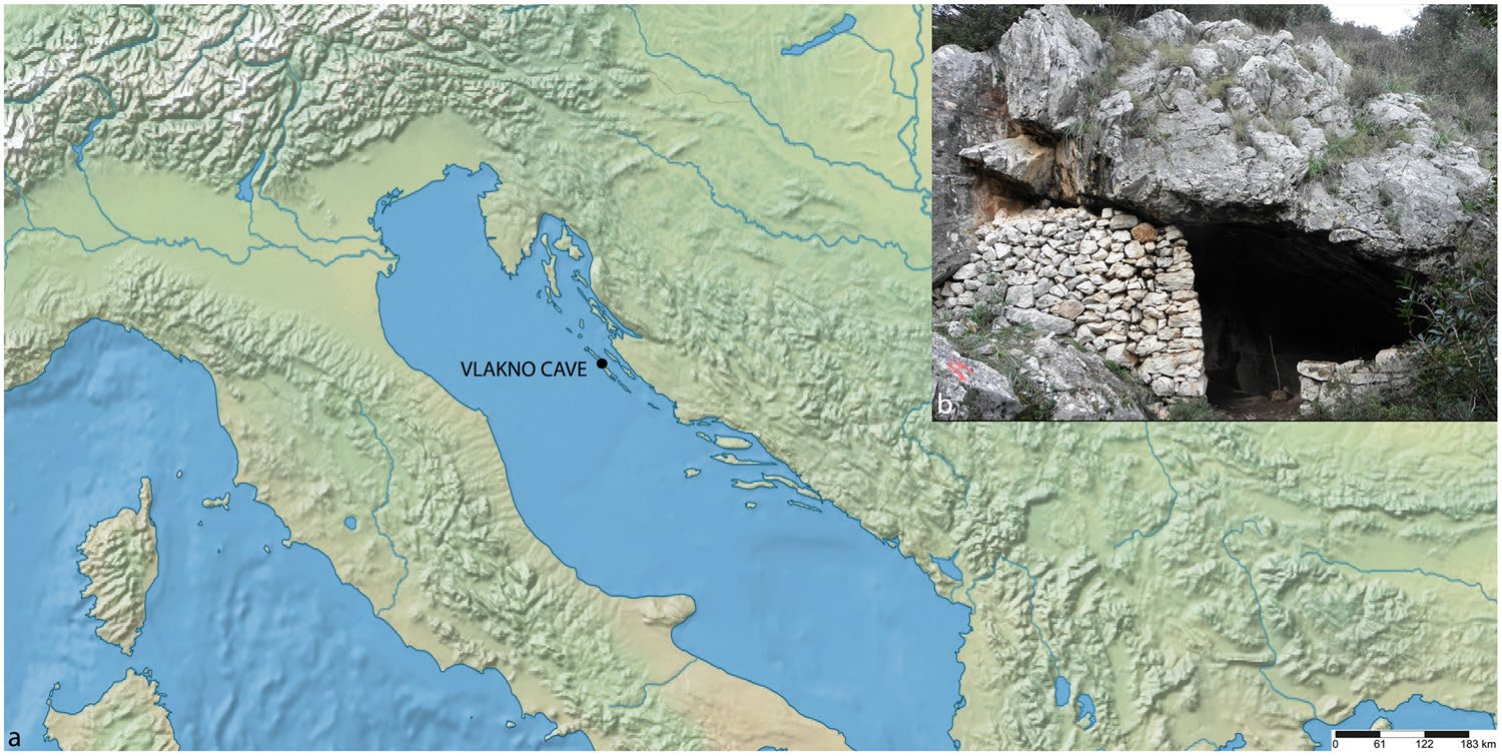
Vlakno site location (**a**) and view of the entrance of the cave (**b**).

In this contribution, we offer new insights into the complexity of Mesolithic diets in the Central Mediterranean by presenting the results of our analysis of dental calculus remains from an individual buried at the site of Vlakno Cave on Dugi Otok (Fig. 1). The analysis of dental calculus presented here will be compared with the results obtained from stable isotope analysis in order to provide an alternative view of Holocene forager dietary and non-dietary behaviour in the Mediterranean Basin as a whole.

The beginning of the Holocene marks a period of profound environmental changes in this part of the Mediterranean, which deeply reshaped the geography of the Adriatic region and the nature of human-environment interactions. To date, attempts to understand forager subsistence dynamics and dietary preferences in this region have focused on traditional, protein-sensitive approaches, such as archaeozoological and stable isotope analysis^10–17,25–31^.

## Archaeological Background

**Figure 2:**
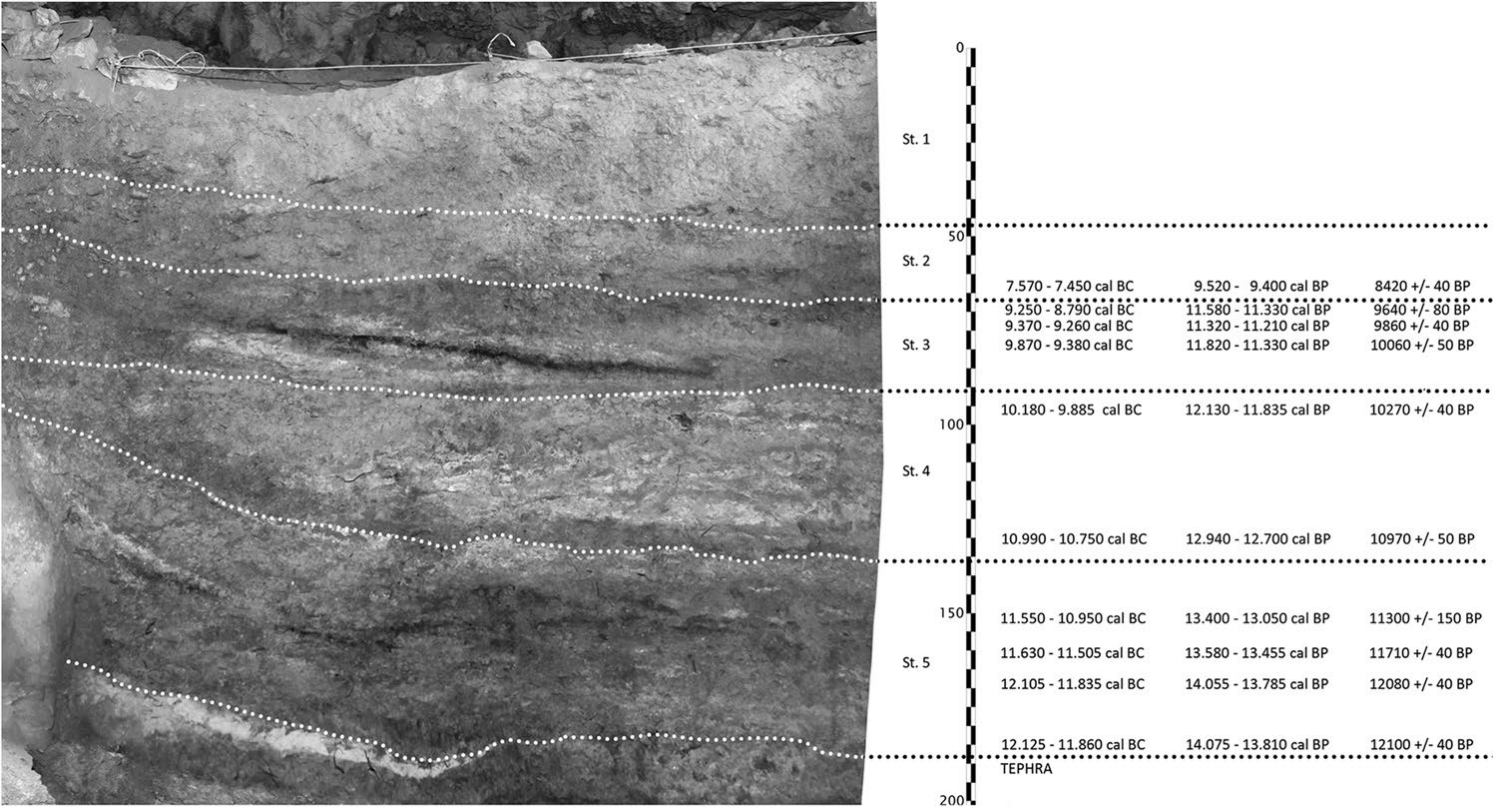
Stratigraphy of the site and chronology.

The site of Vlakno cave is situated on the island of Dugi Otok along the Eastern Adriatic coast of present-day Croatia (Fig. 1). The cave has yielded evidence of Late Upper Palaeolithic (Epigravettian) to Mesolithic hunter-gatherer-fisher occupation^5^. Archaeological investigations at the site started in 2004^32^ while systematic excavations have been on-going since 2011. A 5 m-deep test trench did not reach the bedrock and covers a possibly continuous sequence from *ca*. 18,010–17,560 cal BC at 95% confidence (Beta-302247: 16,330 ± 70 BP). Five main cultural strata (Stratum 1 to 5) have been proposed covering the Epipalaeolithic and Mesolithic periods^33^ (Fig. 2). The upper three strata were deposited during the Holocene. Mesolithic layers start from the surface of Stratum 1 where Mesolithic deposits are mixed with intrusive Neolithic to Medieval artefacts^5^. Mesolithic Strata 2 and 3 roughly correspond with the Boreal and Preboreal chronozones. Based on the radiocarbon measurements, Mesolithic occupation started *ca*. 9880–9370 cal BC at 95% confidence (Beta-30276: 10,060 ± 50 BP)^34^. The chipped stone tool assemblage shows a gradual transition towards a typical Mesolithic assemblage but with strongly pronounced Epigravettian traditions. Similar subtle changes have also been identified in the ornamental assemblage, the worked flints and subsistence strategies^33^.

The analysis of the faunal remains demonstrated a predominance of mammals (88.4% of the total vertebrate assemblage) followed by fish and birds (11.6% of the total vertebrate assemblage)^34^. In the Early Mesolithic (Stratum 3), the most common mammalian taxon was red deer (*Cervus elaphus* L.), followed by red fox (*Vulpes vulpes* L.). In the chronologically later Stratum 2, the red fox is the predominant taxon followed by red deer. Fish remains are present in the faunal assemblage from the beginning of the Mesolithic but there is a significant increase in Stratum 2 (increasing from 1.7% to 9.5% of the total assemblage per stratum)^34^. This difference may correspond with an environmental change, including sea level rise, with Dugi Otok becoming separated from the mainland already before *ca*. 10,194–8351 cal BC at 95% confidence (Z-3660: 9760 ± 280 BP)^35^. Consequently, the local population of red deer may have become vulnerable and overexploited which in turn led to a diversification in subsistence strategies. Fish bones have preliminarily been identified as Sparidae (sea-breams, porgies) and Scombridae (tunas, mackerel). Taxonomically, there is a change in the bird assemblage between the two strata. Wild pigeon (Columbidae) predominates in both, but its frequency decreases in the later deposits, coinciding with an increase in taxa richness. Other taxa are represented by a few bones and include aquatic (cf. rail, duck), steppe (great bustard, *Otis tarda* L.) and some other species (birds of prey, small passerines)^34^. There was also an abundance of mollusc shells. The most abundant are land snails *Helix* sp., showing a slight decrease in their frequency towards the younger Mesolithic levels. The second most abundant group is made up of various taxa of marine gastropods and bivalvia (*Osilinus* sp., *Patella* sp., *Ostrea* sp.) with a pattern of increased diversity in Stratum 2^5^.

**Figure 3:**
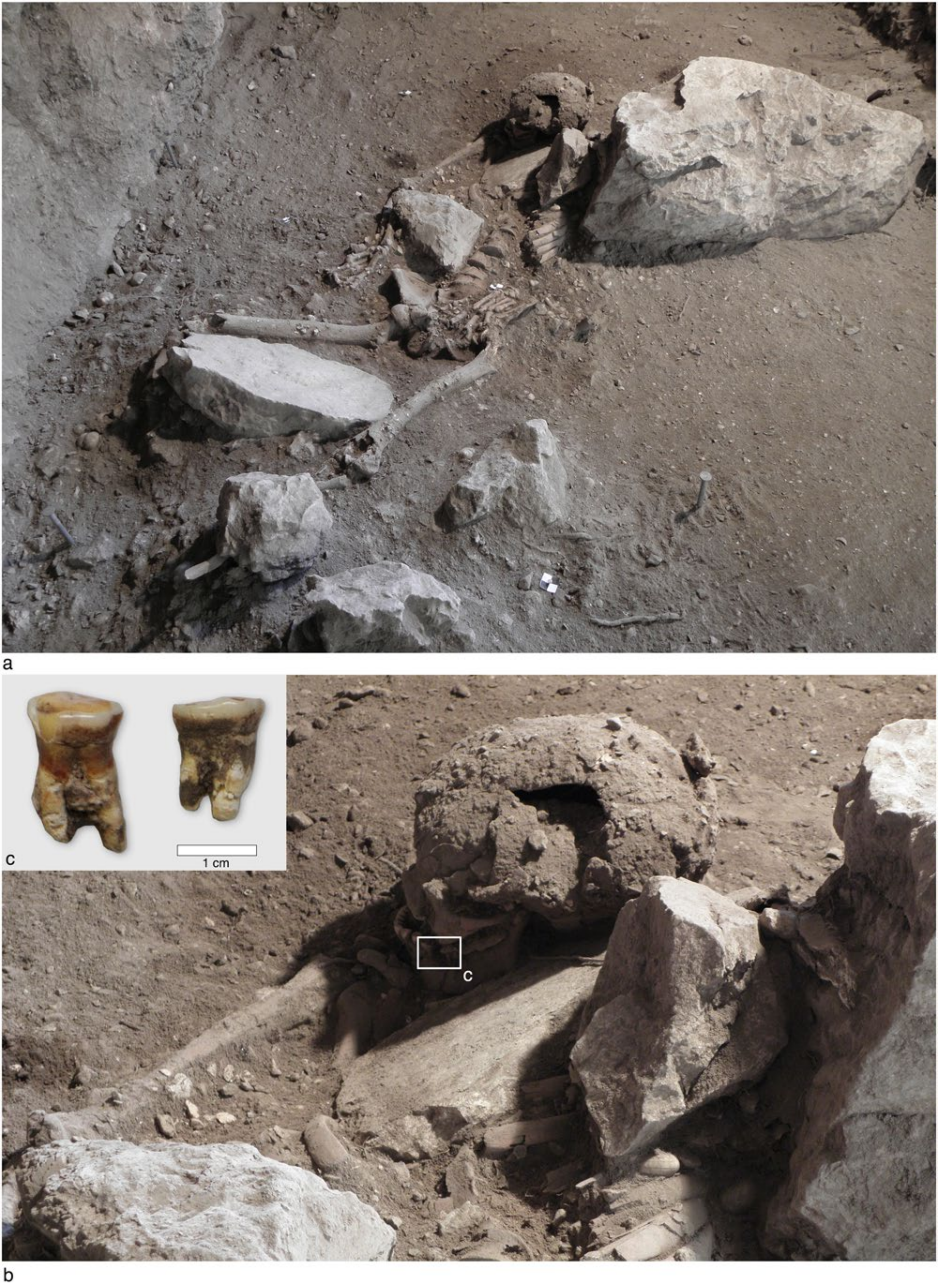
Mesolithic burial from Vlakno cave*:* (**a**) View of the burial; (**b**) Close up of the skull; (**c**) Detail of the teeth and dental calculus.

In Mesolithic Stratum 2, a well-preserved skeleton of an adult male, aged between 30 and 40, was found in an extended supine position (Fig. 3). Paleopathological analyses undertaken according to established criteria described in literature^36,37^ demonstrated mild osteophyte development on the distal right humerus and proximal right ulna suggesting a mild form of degenerative osteoarthritis on the right elbow, as well as marginal osteophyte development on the 2^nd^, 3^rd^, 4^th^ and 5^th^ lumbar vertebrae. Schmorl’s defects were also present on the 1^st^ and 2^nd^ lumbar vertebrae. These defects result from protrusions of the nucleus pulposus of the intervertebral disc through the vertebral body endplate and into the vertebral body. During the excavation, because of the arid soil no traces of a burial pit have been found, but given the age difference between the radiocarbon measurements of Stratum 2 and the burial itself, it must have been dug into the earlier deposits.

The burial was directly AMS radiocarbon (^14^C) dated in two different labs: Beta-311088 measured a phalanx that gave a radiocarbon age of 8420 ± 40 BP, which calibrates to *ca*. 7468–7185 cal BC at 95% confidence; OxA-34518 made on the right proximal ulna shaft fragment yielded a radiocarbon age of 8490 ± 45 BP that calibrates to *ca*. 7591–7496 cal BC at 95% confidence. However, as discussed below, based on the stable isotope values obtained on this individual, the radiocarbon ages are likely to have been affected by a marine reservoir effect. Since the ^14^C results are probably several hundred years too old, the individual most likely falls somewhere towards the end of the 8^th^ millennium BC. At present, in the absence of a ‘perfect pair’ of datable material, which is contemporaneous with the burial but unaffected by a reservoir effect (e.g. the remains of terrestrial herbivores), it is not yet possible to determine the correction factor with certainty. The global marine reservoir age (ΔR) is assumed to be around 400 ^14^C years. A more precise value for calibration purposes is 405 ± 22 ^14^C years^38^ while more refined estimates have recently been suggested for the Eastern Adriatic area depending on the type of sampled marine organism^39^. However, three other Mesolithic radiocarbon measurements from Vlakno fall into the duration of the Early Mesolithic and are at least a 1000 years older than the date obtained on the burial^40^. Presently, it is unclear whether the burial was interred during the Late Mesolithic at an abandoned site with the remains of an earlier Mesolithic occupation, or during the course of occupation in the Late Mesolithic.

## Materials and Methods

### Dental calculus analysis

Dental calculus or tartar is the mineralised biofilm of dental plaque adhering to the tooth enamel, composed primarily of calcium phosphate mineral salts deposited between and within remnants of formerly viable micro-organisms^41^. The continuous production of saliva in the mouth ensures the presence of minerals – key for the formation of calculus^42^. As the deposition of mineralised plaque ceases at the death of an individual with the production of saliva, the calcified plaque represents a sealed repository of unique human biographic information related to the individual’s hygiene, dietary and non-dietary preferences and habits as well as lifestyle^20,22,43,44^. To date, ancient dental calculus analysis has been applied to the study of humans and hominins, even on several million year old fossils, providing insights into their diets and evidence of materials (e.g. wood debris, charcoals and plant fibres) ingested or inhaled while performing daily life activities or using teeth as “third hand” in para-masticatory tasks^20,21,24,45–47^. Recently, it has been emphasised that non-dietary information entrapped in dental calculus has only been partially explored and understood^48^.

Dental calculus was sampled from the well preserved and directly AMS radiocarbon (^14^C) dated skeleton recovered in Stratum 2 (Fig. 3). Mineralised plaque was removed using a metal dental pick. Gloves were worn at all times to reduce contamination. Once removed, the calculus samples were stored in sterile Eppendorf tubes and transported to the laboratory for analysis. Soil still adhering to the surface of the calculus was removed in the laboratory under a microscope. A fine sterile acupuncture needle with 0.06 N HCl was used to gently scrape off very small areas of dirt adhering to the surface. This procedure was essential due to the very small nature of the fragments of calculus available for analysis. This process was conducted using a stereomicroscope at a magnification up to 100x. Once the surface was sufficiently clean, calculus samples were washed in ultrapure water (up to three times) in order to remove any traces of sediment. This method allowed us to remove the dirt from the surface, which minimally affected the calculus whilst ensuring that the surface was completely free of contaminants. Then, the calculus was degraded in a weak solution of 0.06 N HCl in order to extract the microfossils entombed in the calculus matrix following established protocols^44^. The contaminated soil was examined as an additional precaution. The dissolved calculus was mounted on slides using a solution of 50:50 glycerol and ultrapure water. Examination of microfossils in tartar was carried out using Zeiss and Olympus compound polarized microscopes (100x–630x) at the University of York and a Zeiss Imager2 polarized microscope (100x–600x) at Sapienza University of Rome. Starches were identified on the basis of their 3D morphology, presence and shapes of features (lamellae, hilum, bumps and depressions of their surface), characteristics of the extinction cross under polarized light microscopy. The reference collections of modern plants native to the Balkans, the Mediterranean region and Northern Europe housed at the Sapienza University of Rome and the University of York were used for comparison. In addition, samples of modern plants were provided by the Botanical Garden “Jevremovac” of Belgrade, the Genomics Research Centre (CREA) of Fiorenzuola d’Arda and the Agricultural Research Council (CRA) at Sant’Angelo Lodigiano in Italy. The same reference collection has been successfully by the authors in both dental calculus and stone tool analyses^21,22,24,44,49,50^. Previously published literature on local flora was also considered during the analyses. It must be stressed that methodologies and criteria for the identification of archaeological starch granules and microfossils entrapped in dental calculus were also based on the study of renowned literature in the field of modern and ancient starch research^49,51^.

### Stable isotopes analysis

Carbon (δ^13^C) and nitrogen (δ^15^N) stable isotope analyses are routinely undertaken to reconstruct the dietary habits of past populations. Human bone is a complex mixture of organic and inorganic materials that is continually remodelled during a person’s lifetime, and therefore at death the chemistry of the last 10–15 years of life is retained^52^. Since bone is sensitive to contamination and decomposition from the burial environment, hydroxyapatite analyses were not performed. Carbon and nitrogen stable isotopes used in palaeodietary studies are often obtained from bone collagen and primarily relate to the protein content^53^. This creates a bias towards the isotopic recognition of plant foodstuffs in comparison to other dietary resources^54^. δ^13^C values primarily differentiate between marine and terrestrial foodstuffs, and as such a higher δ^13^C value reflects the long-term consumption of marine derived protein. It is widely acknowledged that the marine and terrestrial endpoints are −12 ± 1, and −21 ± 1‰ respectively^19^. It is also possible to distinguish between C_3_ and C_4_ photosynthetic plants on the basis of δ^13^C values. In comparison, δ^15^N is an indicator of the trophic level hierarchy and can differentiate between freshwater organisms and terrestrial mammals as well as plants. δ^15^N values in terrestrial herbivores are generally between 4 and 7‰, while humans or carnivores higher up in the trophic level tend to have δ^15^N values 3 to 4‰ higher than the animals they consume^55^.

### Experimental analysis

The micro-debris found in the dental calculus was compared to a reference collection composed of micro-remains extracted from modern plants native to the Mediterranean, North African regions and Northern Europe. Non-dietary items, which might have been used for crafts or accidentally inhaled as dust during craft and dietary related activities (e.g. wood, plant and animal fibres, feathers, etc.), as suggested elsewhere^48^, have also been considered as reference material. Agate mortars and distilled water were used to grind fresh and dry botanical and non-botanical samples following an established experimental protocol^49,50^, which also included the use of different grinding modalities in order to document distinctive states in the starch granules or changes in size due to the preparation processes. Mortars were carefully cleaned between experiments with soap and hot water for a prolonged period of time. Boiled, ground, and chewed seeds were also considered as experimental reference items. The species relevant to the study are discussed in detail below (see results). Descriptive and interpretative criteria have been chosen according to internationally recognised standards in the field of modern and ancient starch granule research^56^. Finally, due to the fish remains on site and contemporaneous sites in the region^30^ an experimental reference collection of skin, flesh and scales of Mediterranean marine taxa was created. This collection included Atlantic mackerel (*Scomber scombrus* L.), gilthead sea bream (*Sparus aurata* L.), white sea bream (*Diplodus sargus* L.) and European seabass (*Dicentrarchus labrax* L.) that had been processed by a number of methods, including boiling or roasting. In addition, chewed remains were included as well as a range of terrestrial animal tissues, including hair, skin and flesh particles, bone, cartilage, etc. to aid the identification of possible fish and mammalian tissues in tartar.

## Results

### Dental calculus

#### Plant residues

More than 30 starch granules were retrieved from the dental calculus. These were derived from two different morphological types, later assigned to the Triticeae (wheat and barley tribe) potentially to the Poeae (oat tribe) tribes of the Poaceae Families (grasses).

**Figure 4:**
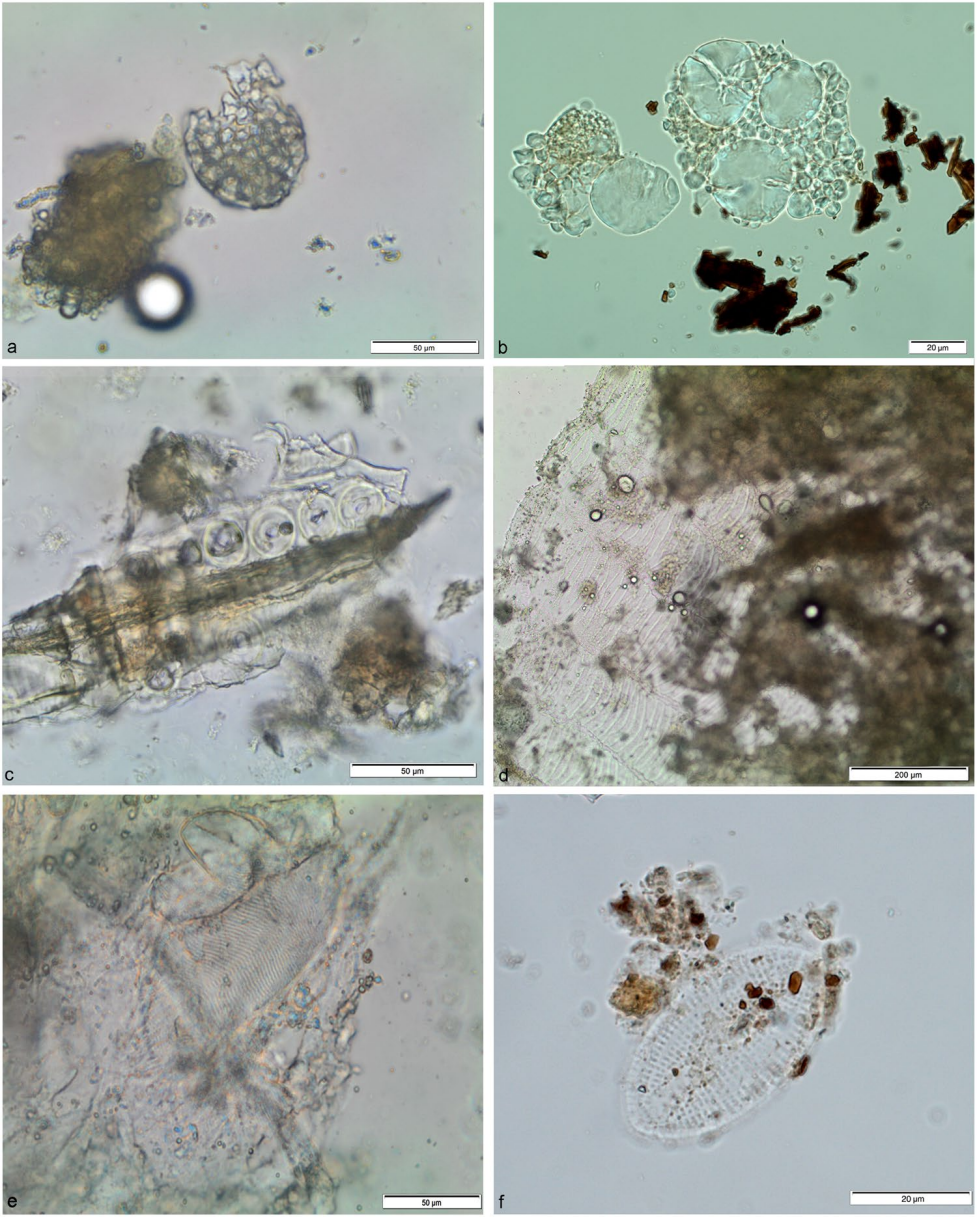
Plant and animal micro-residues identified in Vlakno tartar: (**a**) Lumps of starch granules still embedded in the calculus matrix; (**b**) Starch granules with bimodal distribution; (**c**) Conifer wood fibre; (**d**) Fragment of fish scale entrapped in calculus matrix; (**e**) Fragment of fish flesh; (**f**) *C*. *placentula* diatom.

**Figure 5:**
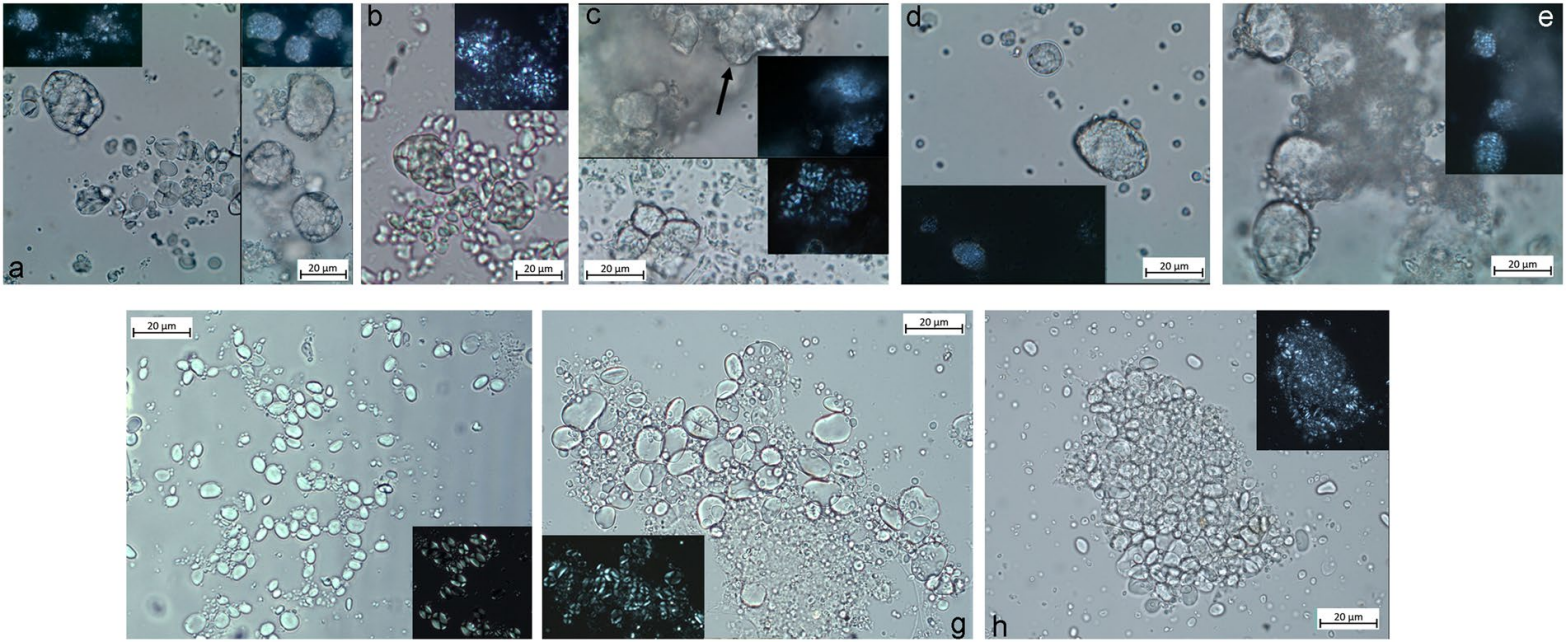
Modern starch granules used as reference: (**a**) *Avena sterilis*; (**b**) *Avena fatua*; (**c**) *Sesleria cerulea*; (**d**) *Phalaris minor*; (**e**) *Phalaris paradoxa*; (**f**) *Hordeum marinum*; (**g**) *Hordeum bulbosum*; (**h**) *Hordeum secalinum*.

Type I. Various small compound granules found in large sub-oval aggregates represent the first and most numerous typology of starch granules found in the Vlakno dental calculus (Fig. 4a). The size and shape of these lumps that were often intact and often still embedded in the calculus matrix, can be compared to those characterising species of many genera of the Poeae Tribe (oats tribe) in our reference collection such as *Avena sterilis* L., *Avena barbata* Link., *Avena fatua* L. and *Phalaris minor* Retz., although other tribes of grasses growing in the Adriatic region of the Balkans, the Sesleriinae (e.g. *Sesleria albicans* Kit., *Sesleria caerulea* L., *Sesleria* Ard., S. *tenuifolia* Schrad., etc.), could not be excluded (Fig. 5a–e).

Type II. The second type of starch granules is characterised by a “bimodal distribution”, which has been shown to be a characteristic of most grasses of the Triticeae tribe (Fig. 4b). These granules were found intact or with some damage but with characteristics still visible (including lamalleae), lodged close together in the amyloplast and calculus matrix. In addition, some charcoal flakes still adhered to them, possibly from smoke in the environment or from roasted food. The bimodal distribution in the archaeological granules was characterised by the co-presence of large and small granules, respectively known as Type A and B. In the Vlakno calculus, the large starch granules (*ca*. 23μm in diameter) were oval to round in 2D and lenticular in 3D. They had a central hilum, deep fissures and no visible lamellae. Their morphology is not completely intact, which is very likely due to some form of processing and/or cooking as well as chewing. The small granules (≤10 μm in diameter) were spherical in shape with a central hilum. Based on our previous work, to which we demand for a detailed discussion^49^, we highlight the following points. We exclude the possibility that the archaeological granules belonged to the *Aegilops* genus as they are mostly spherical and oval and lacked lamellae, which, on the contrary, are visible through the whole starch body of granules belonging to the six species of the *Aegilops* genus represented in the flora of region (*Aegilops cylindrica* (Host.) Ces., *Aegilops geniculata* Roth., *Aegilops lorentii* Hochst., *Aegilops neglecta* Req. ex Bertol., *Aegilops triuncialis* L., and *Aegilops uniaristata* Vis.)^57^. Indeed, the high number of Type B small round granules in our sample as well as the absence of clear lamellae on the body of Type A granules suggest that the identified archaeological starch granules may belong to one of the wild species of barley (*Hordeum* spp.) (e.g. *Hordeum bulbosus* L., *Hordeum marinum* Huds., *Hordeum murinum* L., *Hordeum secalinum* Schreb.), which are commonly found and identified in the flora of the region^58^ (Fig. 5f–h).

**Figure 6:**
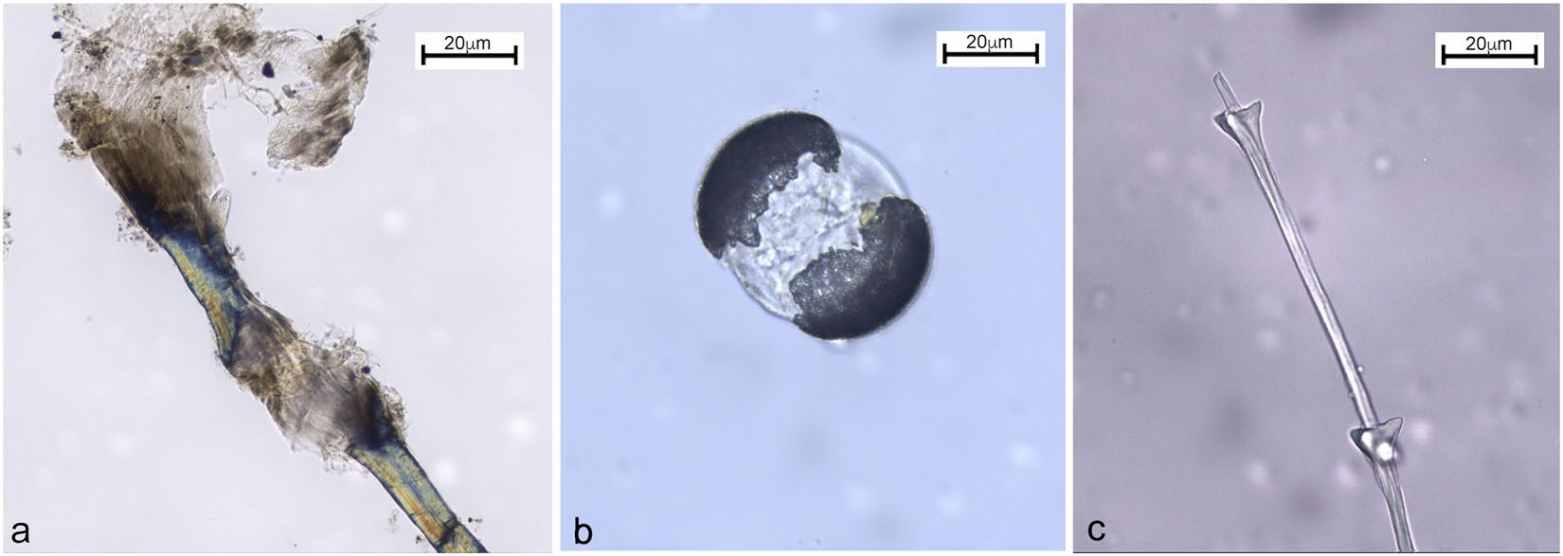
Non-dietary micro-debris identified in archaeological tartar: (**a**) Nettle fibre; (**b**) Conifer pollen; (**c**) Fragment of feather  barbule.

A variety of plant remains not consisting of starch granules were also found. Although a portion was too damaged to be diagnostic, some micro-remains were attributed to a non-dietary origin. In particular, two wood fragments with characteristic conifer tracheid fibres were identified (Fig. 4c, cf. Supplementary Information), and were very similar to those retrieved in a previous study^24^, demonstrating that this kind of evidence does preserve in calculus. Bast fibres still embedded in the calculus matrix were also retrieved (Fig. 6a). When observed under polarised light, these fibres possessed characteristics such as a narrow lumen and a dislocation band, which are known to be a characteristic of plants used for their fibrosity in cordage and textiles (e.g. nettle, flax and hemp)^59^. On the basis of our reference collection, the region and chronological context we suggest that the fibres could belong to nettles (*Urtica* spp.). Two damaged grains of conifer pollen were also identified, supporting the identification of the wood debris as that of conifer (Fig. 6b).

#### Animal micro-remains

**Figure 7:**
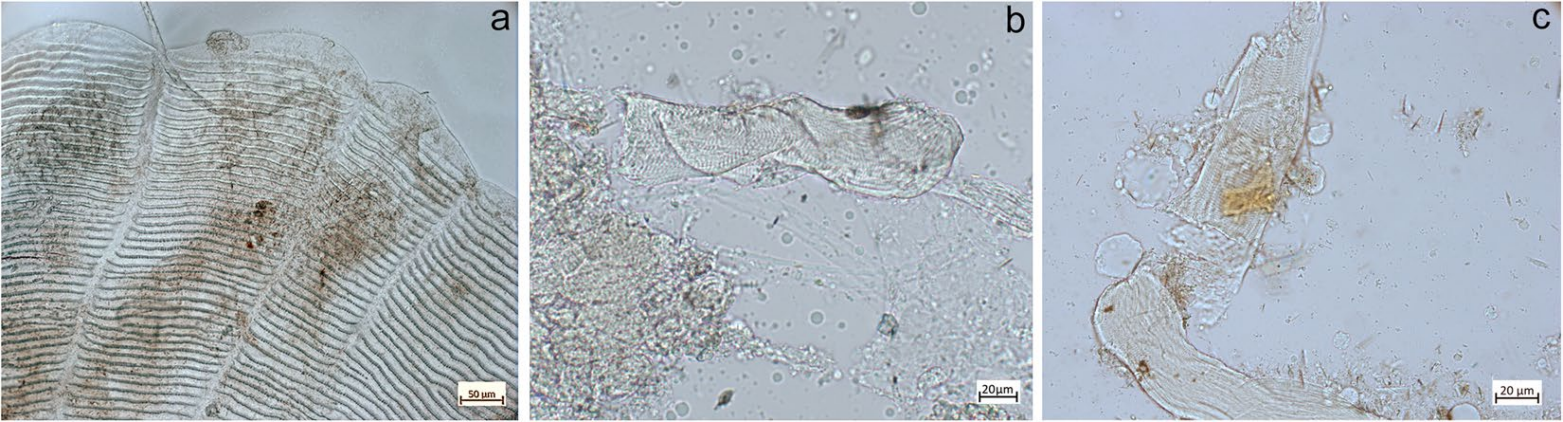
Experimental fish tissues: (**a**) Scale fragment from a gilthead sea bream; (**b**) and (**c**) Fragments of Atlantic mackerel flesh.

Three fragments of fish scale(s) were found lodged *in situ* in the Vlakno calculus (Fig. 4d,e). Although incomplete, the characteristic radii (Fig. 4d) and circuli (Fig. 4e) were clearly visible under polarized light. They were all whitish in colour. While dental calculus fragments can sometimes visually appear similar to a fish scale visually (as they form by apposition of the bacterial concretions) this possibility was excluded by injecting additional HCl to the slide where the remains were found. By doing so, it was possible to further free a part of the scale and observe the dissolution of the calculus adhering to it. Thus, excluding the possibility that the observed structure was a fish scale and not a fragment of calculus. Despite our reference collection, we were unable to identify the scale to the family or species taxonomic levels due to a lack of diagnostic features. That being said, structurally the remains were very similar to the Scombridae (tunas, mackerel) and gilthead sea bream scales (Fig. 7a–c) from our experiments. In addition, at least two fragments of animal micro-debris were also identified and interpreted as fish muscle bands on the basis of comparison with our experimental reference collection. They were white-yellowish in colour and composed of W-shaped parallel fibres often distorted but still adhering to the calculus matrix. To our knowledge, this is the first time that fragments of fish scale(s) and muscle fibres have been identified in dental calculus.

Among the animal micro-fragments, numerous barbule fragments were found in the archaeological calculus and tentatively attributed to the Anatidae family based on their shape, features and distance of their nodes (Fig. 5c and Supplementary Information).

#### Other micro-remains

Two comparable diatoms were recovered entrapped in the calculus matrix (Fig. 4f). The morphological characteristics observed at transmitted light indicate that both remains belong to *Cocconeis placentula* (Ehrenb.) a very widely-distributed diatom found in almost all freshwater or brackish water where the pH is circum-neutral or alkaline. *C. placentula* (Ehrenb.) is very common in benthic habitats, where it attaches to rocks, macrophytes and algae. It is a fast-growing, pioneer species that is able to colonise bare substrates quickly. Although *C. placentula* (Ehrenb.) is found throughout the year, such the diatom is most abundant in rivers in the summer where it can form 80–90% of all individuals present in an epilithic sample.

### Stable isotope analysis

**Figure 8:**
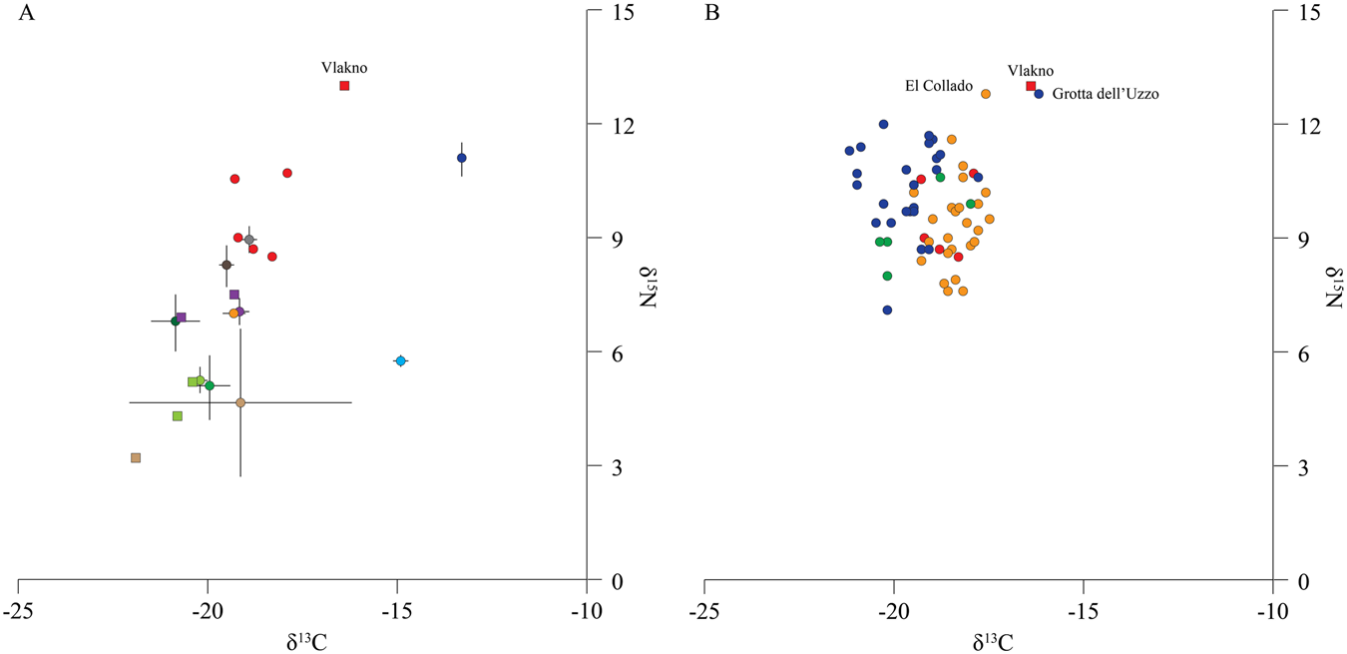
Stable isotope analysis. (**A**) δ^13^C and δ^15^N stable isotope data obtained on human and mammalian bone collagen from Vlakno (squares) compared with data from Mesolithic human and faunal remains (circles) throughout Croatia (data from)^16,27^; (**B**) δ^13^C and δ^15^N stable isotope data obtained on human bone collagen from Vlakno (square) compared with data from 66 Mesolithic and/or Mesolithic-Neolithic humans (circles) from Corsica (green), Croatia (red), Sicily (blue) and Spain (orange) (after)^10–17,25–27,61,76^. Note that the plotted data represents samples that were securely assigned to the Mesolithic, which also had a C:N atomic ratio between 2.9–3.6^77^.

During the AMS radiocarbon (^14^C) dating, the δ^13^C and δ^15^N stable isotope values of −16.1 and 13.0‰ respectively were obtained by the Oxford Radiocarbon Accelerator Unit whilst a δ^13^C isotope value of −16.6‰ was obtained by Beta Analytical. The δ^13^C values are presently some of the highest obtained from human bone collagen dating to the Mesolithic in the Mediterranean basin^14,15,17^, and suggest a significant contribution of marine derived protein. Using a rudimentary linear model^60^ we have estimated that the individual acquired protein of the order of 46.1% from marine organisms. Moreover, the δ^15^N is the highest in the region^14,15,17^ and suggests that the foods consumed were rich in protein. In agreement with Mannino *et al*.^15^ marine organisms higher up in the trophic level hierarchy are likely to have been consumed although terrestrial animals should not be ignored. In order to obtain a dietary baseline for the human interred at Vlakno, we also analysed three different terrestrial mammal species, red deer, hare (*Lepus* sp.) and red fox. These data are listed in Table 1 and presented in Fig. 8. The δ^13^C and δ^15^N stable isotope values were within the range of values obtained previously from Croatia^27^.

**Table 1:** Stable isotope data obtained on the human bone collagen as well as those data obtained from the three terrestrial species (that were measured in duplicate) from the Mesolithic levels of Vlakno Cave. Key: n.d., no data.

Sample	Context	Species	Element	δ^13^C VPDB	δ^15^N AIR	C:N atomic ratio
Beta-311088	Burial 1	*Homo sapiens*	Phalanx	−16.6	n.d.	n.d.
OxA-34518			Ulna, dex.	−16.1	13.0	3.2
1A	Sector East, sq. 15, context 14	*Cervus elaphus*	Metatarsus	−20.5	5.2	3.3
1B				−20.3	5.3	3.3
**1 Mean**				−**20**.**4**	**5**.**2**	**3**.**3**
2A	Sector East, sq. I5, context 14	*Cervus elaphus*	Phalanx I	−20.8	4.3	3.3
2B				−20.7	4.2	3.3
**2 Mean**				−**20**.**8**	**4**.**3**	**3**.**3**
3A	Sector East, sq. I5, context 14	*Vulpes vulpes*	Radius	−20.6	6.6	3.6
3B				−20.8	7.2	3.6
**3 Mean**				−**20**.**7**	**6**.**9**	**3**.**6**
4A	Sector East, sq. I5, context 14	*Vulpes vulpes*	Femur	−19.3	7.4	3.3
4B				−19.4	7.5	3.3
**4 Mean**				−**19**.**3**	**7**.**5**	**3**.**3**
5A	Sector East, sq. I5, context 14	*Lepus* sp.	Tibia	−22.0	3.2	3.4
5B				−21.8	3.2	3.4
**5 Mean**				−**21**.**9**	**3**.**2**	**3**.**4**

## Discussion

In the last 15 years, a growing body of evidence has significantly contributed to our understanding of dietary habits in pre-agrarian societies of the Mediterranean basin. In light of this, the role of marine resources and plant foods still remains unclear due to various methodological hindrances: a focus on protein-sensitive methods, the poor preservation of plant and fish remains, and the lack of systematic recovery techniques for organic residues. This ambiguity is particularly significant for forager groups who lived in close proximity to the coast or on the islands of the Central Mediterranean^7,61–63^.

At the beginning of the Holocene, a wide range of available vegetal resources became available due to the newly established environmental conditions in the Eastern Adriatic region^64–66^. While the recovery of two different group of plants from one individual is suggestive that carbohydrate-rich resources were   a part of the dietary habits of the Mesolithic forager from Vlakno, understanding how   different plant foods contributed to the Mesolithic diet of the Vlakno group is challenging as no direct correlation can still be drawn between the presence of plant micro-remains in ancient calculus, the quantity of plant foods that entered the diet and the moment those were consumed by the individual^23^. Yet, botanical microfossils recovered in the archaeological dental calculus seem to strongly suggest different species of plants were selected and consumed by the individual and potentially local foragers as a part of their diet and culinary tradition. Besides the botanical remains trapped in Vlakno calculus, direct archaeological evidence for the consumption of plant foods in the Adriatic region during the Mesolithic is restricted to the site of Vrbička Cave in Montenegro where fragments of hazelnut shells were recovered from the Late Mesolithic layers after extensive flotation^67^. To the south, the remains of pears and a few peas since *ca*. 7300 cal BC and wild oats and barley after *ca*. 7000 cal BC are known from the site of Franchti Cave in Greece^68^. On the other side of the Adriatic, oats (*Avena* sp.) have been consumed since the Middle Upper Palaeolithic. At the site of Apulia in Italy, starch granules and phytoliths have been identified on Gravettian ground stone tools at the cave site of Paglicci^69^. During the Early Holocene data concerning the consumption of plant resources is derived from Grotta di Pozzo in the Fucino Basin of Abruzzo where carpological remains of bearberry, cornelian cherry and wild grapes were recovered^70^. Lastly, the possible consumption of plant foods is also available for the Mesolithic foragers at Grotta dell’Uzzo in Sicily based on the stable isotope data^13,14^.

While animal micro-residues have rarely been found in the course of dental calculus microfossil analysis, the analysis of the Vlakno individual yielded the exceptionally well-preserved fragments of fish flesh and scales. This was possible due to the anatomy of the fish scale, which may have provided an ideal substrate for the bacterial to grow over and above it, as demonstrated by the need of adding further HCl to the slide to free the scale to permit identification. Such evidence is unique and emphasises the potential of ancient tartar to capture other lines of evidence than plant remains. While we cannot confidently confirm when flesh and scale(s) fragments were entrapped in the tartar, the abundance of fish remains in the Mesolithic layers of Vlakno as well as the stable isotope data demonstrate that marine resources were regularly consumed by the individual; alternatively, de-scaling activities using teeth may have taken place. Tartar formation depends on different factors and can vary from one individual to another. While it was not possible to identify the scale to the family or species taxonomic levels, abundant Atlantic chub mackerel (*Scomber colias* Gmelin.), gilthead sea bream and cuttlefish remains have been recovered in the Mesolithic layers of Vlakno cave^34^. The abundance of fish remains from the Mesolithic layers at the site demonstrates that Vlakno cave may have been used seasonally for fishing^5^. Not far from Vlakno cave, the site of Vela Spila on the Island of Korčula yielded numerous fish remains^30^. The assemblage was dominated by the cf. Atlantic chub mackerel (*Scomber colias*), and it was suggested that the specialised capture and processing of this species took place during the Mesolithic and Neolithic periods. Although no comparison can be made between the numerous fish remains recovered from Mesolithic sites in the westernmost part of the Mediterranean^71^ or Northern Europe and the Atlantic^72^, at Vela Spila nearly half a ton of Atlantic chub mackerel was processed during the Early Mesolithic^30^. Moreover, additional evidence relating to fishing in the Central Mediterranean derives from Sicily where at Grotta dell’Uzzo and Grotta d’Oriente the archaezoological remains document an increase in the exploitation of fish throughout the Mesolithic and across the Mesolithic-Neolithic transition^73^.

The stable isotope values obtained from the Vlakno individual corroborate the data obtained from the analyses of the dental calculus and animal remains. As such these data demonstrate that protein-rich foods (probably marine resources alongside C_3_ terrestrial animals) were consumed. Previous stable isotope analyses in the Eastern Adriatic region suggests that terrestrial resources, particularly red deer and wild boar (*Sus scrofa* L.), were preferentially consumed during the Mesolithic even at coastal localities^15,27^. There are only a few examples that demonstrate the consumption of marine derived protein, a long bone from El Collado (δ^13^C = –17.6‰, δ^15^N = 12.8‰), and a cranium from the site of Grotta dell’Uzzo (δ^13^C = –16.2‰, δ^15^N = 12.8‰)^15,26^. Whilst the current sample size is based on one individual and possibly unrepresentative, it differs from the previously noted Mediterranean Basin-wide trend of predominately terrestrial-based diets during the Mesolithic^74,75^, and which was recently also emphasised for the Mesolithic of Sardinia, Sicily and the Egadi islands^9,12,14^.

Microfossils below 20μm in size, demonstrate the potential use of plant fibres for non-dietary purposes, (e.g. the masticatory apparatus). For instance, portions of wood fragments and xylem tissue are not edible parts of plants, as noted previously^24^. Both bast fibres and wood are potentially the remains resulting from non-dietary activities, which may have included holding, softening or shredding plant fibres for cordage, textile, basketry, net-making and the use of tooth picks, wooden culinary tools, the exposure to smoke from fireplaces as well as dust generated while plucking birds or other crafts. The use of teeth as a “third hand” in para-masticatory activities and oral hygiene may also be possible explanations for the presence of non-alimentary dust in the tartar. Diatoms were also identified in the calculus matrix, which may have derived from a freshwater source in the vicinity of the cave, which the individual had exploited. The recovery of these micro-residues in the calculus matrix confirms the potential of tartar to act as an archaeological repository of environmental as well as occupational debris^48^.

## Conclusions

This contribution details an osteobiography of an adult male individual from a lone Mesolithic burial found in Vlakno Cave on the Island of Dugi Otok in the Croatian archipelago. As our data refer to a single individual they are not expected to provide a comprehensive picture of dietary habits of the period and region. However, our presentation of dietary information available for this individual based on the study of dental calculus along with stable isotope data, and contextualised with the existing archaeological data, provides a significant insight into the lifeway’s of Adriatic and Mediterranean Holocene foragers. Methodologically, we have also shown the potential of dental calculus to provide not only plant-related evidence but also to contribute to the reconstruction of non-dietary related practices of the everyday. By integrating two different strands of data—the analysis of microfossils entombed in dental calculus and the stable isotope analysis of bone collagen—we were able to document how carbohydrate-rich plants and marine resources contributed to Mesolithic food ways. While the data presented in this study remains limited, it potentially demonstrates that Mesolithic dietary strategies in the Eastern Adriatic may have differed from areas in the central and western parts of the basin.

### Statement

No experiments on live vertebrats have been performed for the study. All methods reported in the paper were carried out in accordance with relevant guidelines and regulations.

## Electronic supplementary material

Supplementary Information

## References

[1] Vigne, J.-D. & Desse-Berset, N. *The exploitation of animal resources in the Mediterramean Islands during the Pre-Neolithic*: *the example of Corsica*. (Oxbow Books, 1995).

[2] Čečuk, B. & Radić, D. *Vela spila: višeslojno pretpovijesno nalazište; Vela Luka-otok Korčula*. (Centar za kulturu ‘Vela Luka’, 2005).

[3] Martini, F. in *La cultura del morire nelle società preistoriche e protostoriche italiane*. *Studio interdisciplinare dei dati e loro trattamento informatico*. (ed. Martini, F.) **3**, 67–86 (Origines, Progetti, 2006).

[4] Radić D (2005). Vela spila: preliminarna analiza starijeneolitičkih i mezolitičkih naslaga iz sonde istražene 2004. godine. Opusc. Archaeol. Rad. Arheol. zavoda.

[5] Vujević D, Bodružić M (2013). Mesolithic communities of Vlakno cave. *Diadora Glas*. Arheol. muzeja u Zadru.

[6] Komšo D (2008). Mezolitik u Hrvatskoj. Opusc. Archaeol. Rad. Arheol. zavoda.

[7] Mannino, M. & Richards, M. The role of aquatic resources in ‘Italian’ hunter-gatherer subsistence and diets. in *Palaeolithic Italy*. *Advanced studies on early human adaptation in the* Apennine Peninsula (eds Borgia, V. & Cristiani, E.) (Sidestone Press).

[8] Courtaud, P., Petersen, H. C., Zemour, A., Leandri, F. & Cesari, J. The Mesolithic burial of Campu Stefanu (Corsica, France). in *Mesolithic Burials-Rites*, *symbols and social organisation of early postglacial communities* 719–731 (2016).

[9] Floris, R., Melis, R. T., Mussi, M., Palombo, M. R. & Iacumin, P. La presenza umana nella Sardegna centro occidentale durante l’Olocene antico: il sito di S’Omu e S’Orku, Arbus, VS. *Atti della XLIV Riun*. *Sci*. *la Preist*. *e la protostoria della Sardegna Cagliari*, *Barumini*, *Sassari 23-28 novembre 2009*, *vol*. *2–3 Comun*. *vol*. *4 Posters*. 999–1004 (2012).

[10] de Pablo JF-L (2013). Late Mesolithic burials at Casa Corona (Villena, Spain): direct radiocarbon and palaeodietary evidence of the last forager populations in Eastern Iberia. J. Archaeol. Sci..

[11] Goude G (2017). New Insights into Mesolithic Human Diet in the Mediterranean from Stable IsotopeAnalysis: The Sites of Campu Stefanu and Torre d’Aquila, Corsica. Int. J. Osteoarchaeol..

[12] Mannino MA (2011). Upper Palaeolithic hunter-gatherer subsistence in Mediterranean coastal environments: an isotopic study of the diets of the earliest directly-dated humans from Sicily. J. Archaeol. Sci..

[13] Mannino MA, Thomas KD, Leng MJ, Di Salvo R, Richards MP (2011). Stuck to the shore? Investigating prehistoric hunter-gatherer subsistence, mobility and territoriality in a Mediterranean coastal landscape through isotope analyses on marine mollusc shell carbonates and human bone collagen. Quat. Int..

[14] Mannino MA (2012). Origin and diet of the prehistoric hunter-gatherers on the Mediterranean island of Favignana (Ègadi Islands, Sicily). PLoS One.

[15] Mannino MA (2015). Climate-driven environmental changes around 8,200 years ago favoured increases in cetacean strandings and Mediterranean hunter-gatherers exploited them. Sci. Rep..

[16] Paine, C., O’Connell, T. & Miracle, P. T. Stable isotopic reconstruction of Early Mesolithic diet at Pupicina Cave. *Mesolith*. *Horizons*. *Oxford Oxbow Books* 210–216 (2009).

[17] Salazar-Garcia DC (2014). Isotope evidence for the use of marine resources in the Eastern Iberian Mesolithic. J. Archaeol. Sci..

[18] Fischer A (2007). Coast–inland mobility and diet in the Danish Mesolithic and Neolithic: evidence from stable isotope values of humans and dogs. J. Archaeol. Sci..

[19] Richards MP, Hedges REM (1999). Stable isotope evidence for similarities in the types of marine foods used by Late Mesolithic humans at sites along the Atlantic coast of Europe. J. Archaeol. Sci..

[20] Hardy K (2016). Dental calculus reveals potential respiratory irritants and ingestion of essential plant-based nutrients at Lower Palaeolithic Qesem Cave Israel. Quat. Int..

[21] Radini, A., Nikita, E. & Shillito, L. M. Human dental calculus and a medieval urban environment. *Everyday life Mediev*. *Eur*. *Environ*. *artefactual approaches to Dwell*. *T*. *country*. *Belgium Brepols*. 10.1073/pnas1018116108 (2016).

[22] Buckley S, Usai D, Jakob T, Radini A, Hardy K (2014). Dental calculus reveals unique insights into food items, cooking and plant processing in prehistoric central Sudan. PLoS One.

[23] Leonard C, Vashro L, O’Connell JF, Henry AG (2015). Plant microremains in dental calculus as a record of plant consumption: A test with Twe forager-horticulturalists. J. Archaeol. Sci. Reports.

[24] Radini A (2016). Neanderthals, trees and dental calculus: new evidence from El Sidrón. Antiquity.

[25] Francalacci P (1988). Comparison of archaeological, trace element, and stable isotope data from two Italian coastal sites. Riv. di Antropol..

[26] Garcia Guix E, Richards M, Subir ME (2006). Palaeodiets of humans and fauna at the Spanish Mesolithic site of El Collado. Curr. Anthropol..

[27] Lightfoot E, Boneva B, Miracle PT, Šlaus M, O’connell TC (2011). Exploring the Mesolithic and Neolithic transition in Croatia through isotopic investigations. Antiquity.

[28] Miracle, P. & Forenbaher, S. Prehistoric herders of northern Istria: the archaeology of Pupićina cave/Pretpovijesni stočari sjeverne Istre: arheologija Pupićine peći. (2006).

[29] Miracle P, Galanidou N, Forenbaher S (2000). Pioneers in the hills: early Mesolithic foragers at Šebrn Abri (Istria, Croatia). Eur. J. Archaeol..

[30] Rainsford C, O’Connor T (2014). & Miracle, P. Fishing in the Adriatic at the Mesolithic–Neolithic transition: Evidence from Vela Spila, Croatia. Environ. Archaeol..

[31] Vika E, Theodoropoulou T (2012). Re-investigating fish consumption in Greek antiquity: results from δ 13 C and δ 15 N analysis from fish bone collagen. J. Archaeol. Sci..

[32] Brusić ZPV (2005). (Vlakno Cave). Hrvat. Arheol. godišnjak.

[33] Cvitkušić, B., Radović, S. & Vujević, D. Changes in ornamental traditions and subsistence strategies during the Palaeolithic-Mesolithic transition in Vlakno cave. *Quat*. *Int*. (2017).

[34] Radović, S., Spy-Marquez, P. & Vujević, D. A tale of foxes and deer or how people changed their eating habitats during the Mesolithic al Vlakno cave (Croatia). in *Holocene Foragers in Europe and Beyond (Papers Presented the Ninth International Conference on the Mesolithic in Europe* MESO *2015*, *Belgrade*, *Serbia)* (eds Borić, D., Antonović, D., Mihailović, B. & Stefanović, S.) (Serbian Archaeological Society, 2015).

[35] Surić, M. Maša Surić, Promjene u okolišu tijekom mlađeg pleistocena i holocena – zapisi iz morem potopljenih siga istočnog Jadrana (disertacija). (University of Zagreb, 2006).

[36] Ortner, D. J. *Identification of pathological conditions in human skeletal remains*. (Academic Press, 2003).

[37] Aufderheide, A. C., Rodriguez-Martin, C. & Langsjoen, O. *The Cambridge encyclopedia of human paleopathology*. **478**, (Cambridge University Press Cambridge, 1998).

[38] Reimer PJ (2013). IntCal13 and Marine13 radiocarbon age calibration curves 0–50,000 years cal BP. Radiocarbon.

[39] Faivre S, Bakran-Petricioli T, Barešić J, Horvatinčić N (2015). New data on marine radiocarbon reservoir effect in the Eastern Adriatic based on pre-bomb marine organisms from the intertidal zone and shallow sea. Radiocarbon.

[40] Vujević, D. & Bodružić, M. In *Holocene Foragers in Europe and Beyond (Papers Presented the Ninth International Conference on the Mesolithic in EuropeMESO 2015*, *Belgrade*, *Serbia)(*eds Borić, D., Antonović, D., Mihailovic, D. & Stefanović, S.) (Belgrade: Serbian Archaeological Society).

[41] Lieverse AR (1999). Diet and the aetiology of dental calculus. Int. J. Osteoarchaeol..

[42] Cowley, G. & MacPhee, T. *Essentials of Periodontology and Periodontics* (Blackwell, 1975).

[43] Hardy K (2009). Starch granules, dental calculus and new perspectives on ancient diet. J. Archaeol. Sci..

[44] Warinner C (2014). Pathogens and host immunity in the ancient human oral cavity. Nat. Genet..

[45] Blatt SH, Redmond BG, Cassman V, Sciulli PW (2011). Dirty teeth and ancient trade: evidence of cotton fibres in human dental calculus from Late Woodland, Ohio. Int. J. Osteoarchaeol..

[46] Blondiaux J, Charlier P (2008). Palaeocytology in skeletal remains: microscopic examination of putrefaction fluid deposits and dental calculus of skeletal remains from French archaeological sites. Int. J. Osteoarchaeol..

[47] Hardy K (2012). Neanderthal medics? Evidence for food, cooking, and medicinal plants entrapped in dental calculus. Naturwissenschaften.

[48] Radini A, Nikita E, Buckley S, Copeland L, Hardy K (2017). Beyond food: The multiple pathways for inclusion of materials into ancient dental calculus. Am. J. Phys. Anthropol..

[49] Cristiani E, Radini A, Edinborough M, Borić D (2016). Dental calculus reveals Mesolithic foragers in the Balkans consumed domesticated plant foods. Proc. Natl. Acad. Sci..

[50] Lucarini G, Radini A, Barton H, Barker G (2016). The exploitation of wild plants in Neolithic North Africa. Use-wear and residue analysis on non-knapped stone tools from the Haua Fteah cave, Cyrenaica, Libya. Quat. Int..

[51] Madella M, Lancelotti C, Garcia-Granero JJ (2016). Millet microremains:an alternative approach to understand cultivation and use of critical crops in Prehistory. Archaeol. Anthropol. Sci..

[52] Richards MP, Fuller BT, Sponheimer M, Robinson T, Ayliffe L (2003). Sulphur isotope measurements in archaeological samples: some methodological considerations. Int J Osteoarchaeol.

[53] Krueger, H. W. & Sullivan, C. H. In S*tab*le Isoto*pe*s in *Nutrition*. *ACS Symposium Series 258* (eds Turnlund, J. & Johnson, P.) 205–222 (ACS Publications, 1984).

[54] Müldner G, Richards MP (2005). Fast or feast: reconstructing diet in later medieval England by stable isotope analysis. J. Archaeol. Sci..

[55] Schoeninger MJ, DeNiro MJ (1982). Carbon isotope ratios of apatite from fossil bone cannot be used to reconstruct diets of animals. Nature.

[56] Torrance, R. & Barton, H. *Ancient Starch Research*. (Left Coast Press, 2007).

[57] Nikolić, T. Flora Croatica Database. Department of Botany, Faculty of Science, University of Zagreb (2008).

[58] Ljustjna, M. & Vitas, B. *Zbornik istrazivackih radova Udruge studenata - Biologije - ‘BIUS’ u Parku prirode Telascica*. (BIUS, 2002).

[59] Bergfjord C, Holst B (2010). A procedure for identifying textile bast fibres using microscopy: Flax, nettle/ramie, hemp and jute. Ultramicroscopy.

[60] Arneborg J (1999). Change of diet of the Greenland Vikings determined from stable carbon isotope analysis and 14C dating of their bones. Radiocarbon.

[61] Bocherens, H. Neanderthal dietary habits: review of the isotopic evidence. *Evol*. *Hominin diets* 241–250 (2009).

[62] Jones, M. Moving north: archaeobotanical evidence for plant diet in Middle and Upper PaleolithicEurope. *Evol*. *Hominin Diet*s 171–180 (2009).

[63] Richards, M. P. 20. Stable isotope evidence for European Upper Paleolithic human diets. *Hublin*, *J*.*-J*., *Richards*, *MP(Eds*.*)*, *Evol*. *Hominid Diets Integr*. *Approaches to Study Palaeolithic Subsist*. *Vertebr*. *Paleobiol*. *Paleoanthropology*. *Springer*, *Dordr*. 251–257 (2009).

[64] Andrič M (2008). Late quaternary vegetation and hydrological change at Ljubljansko barje (Slovenia). Palaeogeogr. Palaeoclimatol. Palaeoecol..

[65] Favaretto S, Asioli A, Miola A, Piva A (2008). Preboreal climatic oscillations recorded by pollen and foraminifera in the southern Adriatic Sea. Quat. Int..

[66] Rossignol-Strick M (1999). The Holocene climatic optimum and pollen records of sapropel 1 in the eastern Mediterranean, 9000–6000BP. Quat. Sci. Rev..

[67] Borić, D. *et al*. In *Holocene Foragers in Europe and Beyond (Papers Presented the Ninth International Conference on the Mesolithic in EuropeMESO 2015*, *Belgrade*, *Serbia(*eds Borić, D., Antonović, D., Mihailovic, D. & Stefanović, S.) (Belgrade: Serbian Archaeological Society).

[68] Hansen J, Renfrew JM (1978). Palaeolithic–neolithic seed remains at Franchthi Cave, Greece. Nature.

[69] Lippi MM, Foggi B, Aranguren B, Ronchitelli A, Revedin A (2015). Multistep food plant processing at Grotta Paglicci (Southern Italy) around 32,600cal BP. Proc. Natl. Acad. Sci..

[70] Lubell D (1999). Exploitation of seasonal resources in the mountains of Abruzzo (central Italy): Epigravettian to Neolithic. *L’Europe des derniers Chass*. Epipaléolithique Mésolithique.

[71] Morales-Muñiz, A. & Roselló-Izquierdo, E. Twenty thousand years of fishing in the Strait. *Hum*. *Impacts Anc*. *Mar*. *Ecosyst*. *A Glob*. *Perspect*. *Ed*. *by Rick*, *TC Erlandson*, *JM*, *Univ*. *Calif*. *Press*. *Berkeley* 243–277 (2008).

[72] Enghoff, I. B. *Regionality and biotope exploitation in Danish Ertebølle and adjoining periods*. (Det Kongelige Danske Videnskabernes Selskab, 2011).

[73] Tagliacozzo, A. *Archeozoologia della Grotta dell’Uzzo*, *Sicilia: da un’economia di pesca ed allevamento*. (Ist. poligrafico e zecca dello stato, 1993).

[74] Schulting, R. Sweet or salty? Isotopic evidence for the use of aquatic resources in Mesolithic Europe (2015).

[75] Schulting, R. & Borić, D. in *The Neolithic of Europe: Papers in Honour of* Alasdair Whittle (eds Bickle, P., Cummings, V., Hofmann, D. & Pollard, J.) 82–105 (Oxbow Books, 2017).

[76] Pouydebat, E. Approche biogéochimique de l’alimentation humaine dans le site prénéolithique du Monte Leone (VIIIe millénaire av J.-C.; Bonifacio Corse-du-Sud). *Univ*. *Paris I*, *Paris* (1997).

[77] DeNiro MJ (1985). Postmortem preservation and alteration of *in vivo* bone collagen isotope ratios in relation to palaeodietary reconstruction. Nature.

