# Supplementary material for: Dental calculus and isotopes provide direct evidence of fish and plant consumption in Mesolithic Mediterranean
Source: Sci Rep. 2018 May 25;8:8147. doi: 10.1038/s41598-018-26045-9 (PMC5970156; doi:10.1038/s41598-018-26045-9)
Supplement: Supplementary file 1 — Supplementary Information [file 41598_2018_26045_MOESM1_ESM.pdf]

Supplementary Information for Scientific Report

Dental calculus and isotopes provide direct evidence of fish and plant consumption in Mesolithic Mediterranean

Emanuela Cristiani[1], Anita Radini[2], Dušan Borić[3], Harry K. Robson[2], Isabella Caricola[1], Maria Letizia Carra[1], Giuseppina Mutri[1], Gregorio Oxilia[1], Andrea Zupancich[1], Mario Šlaus[4], and Dario Vujević[5]

[1]Department of Oral and Maxillo Facial Sciences. "Sapienza" University of Rome. Via Caserta 6, 00161, Rome, (Italy), (Corresponding author, email: emanuela.cristiani@uniroma1.it)

[2]BioArCh, Department of Archaeology, University of York, York YO10 5YW, United Kingdom

[3]The Italian Academy for Advanced Studies in America, Columbia University, 1161 Amsterdam Avenue, New York NY 10027, USA

[4]Anthropological Center, Croatian Academy of Sciences and Arts, 10000 Zagreb, Croatia

[5]Department of Archaeology, University of Zadar, Zadar, Croatia

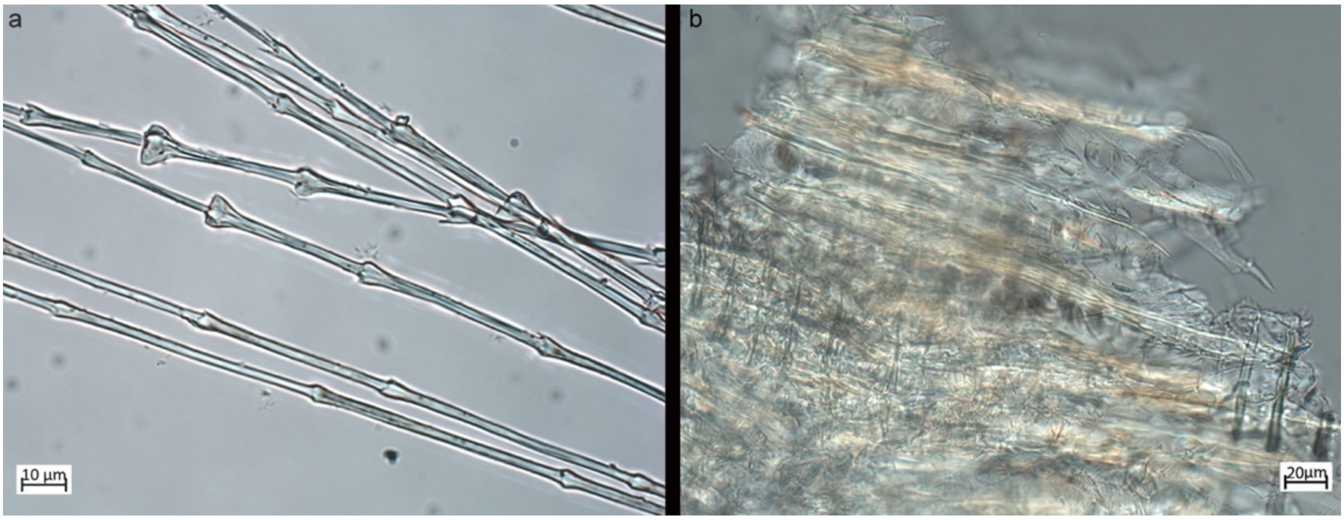

Experimental residues – a. conifer wood; b and c. *Anas platyrhynchos* (mallard) feather.

Site name	Skeletal element	δ^{13}C	δ^{15}N	C:N atomic ratio	Lab nr.	Radiocarbon age ± error (BP)	Period	Country	Reference
Araguina Sennola		-18.8	10.6	n.d.			Mesolithic	Corsica	Bocherens 1999
Casa Corona		-18.5	11.6	3.4	OxA-V-2392-92	7116 ± 32	Mesolithic	Spain	Fernández-López de Pablo et al. 2013
Casa Corona		-19.3	8.4	3.4	Beta-272856	7070 ± 40	Mesolithic	Spain	Fernández-López de Pablo et al. 2013
Uzzo Cave		-21.0	10.4	3.6			Mesolithic	Sicily	Francalacci 1989
Uzzo Cave		-21.0	10.7	3.6			Mesolithic	Sicily	Francalacci 1989
El Collado	Long bone	-17.6	10.2	3.2			Mesolithic	Spain	Garcia Guixe et al. 2006
El Collado	Long bone	-17.6	12.8	3.4			Mesolithic	Spain	Garcia Guixe et al. 2006
El Collado	Long bone	-17.9	8.9	3.4			Mesolithic	Spain	Garcia Guixe et al. 2006
El Collado	Long bone	-18.1	10.4	3.3			Mesolithic	Spain	Garcia Guixe et al. 2006
El Collado	Long bone	-18.2	10.6	3.3			Mesolithic	Spain	Garcia Guixe et al. 2006
El Collado	Long bone	-18.2	10.9	3.3			Mesolithic	Spain	Garcia Guixe et al. 2006
El Collado	Long bone	-19.0	9.5	3.5			Mesolithic	Spain	Garcia Guixe et al. 2006
El Collado	Long bone	-19.1	8.9	3.3			Mesolithic	Spain	Garcia Guixe et al. 2006
El Collado	Long bone	-19.5	10.2	3.4			Mesolithic	Spain	Garcia Guixe et al. 2006
Torre d’Aquila	Radius, dex.	-20.2	8.0	3.4	S-ANU 38011	8715 ± 50	Mesolithic	Corsica	Goude et al. 2017
Torre d’Aquila	1st Molar	-20.2	8.9	3.3	S-ANU 38010	8790 ± 50	Mesolithic	Corsica	Goude et al. 2017

Torre d'Aquila	Long bone	-20.3	8.1	3.5			Mesolithic	Corsica	Goude et al. 2017
Campu Stefanu	Long bone	-20.4	8.9	3.2			Mesolithic	Corsica	Goude et al. 2017
Vela Spilja-Vela Luka	Rib	-17.9	10.7	3.2			Mesolithic	Croatia	Lightfoot et al. 2011
Vela Spilja-Vela Luka	Rib	-18.3	8.5	3.2			Mesolithic	Croatia	Lightfoot et al. 2011
Vela Spilja-Vela Luka	Rib	-18.8	8.7	3.2			Mesolithic	Croatia	Lightfoot et al. 2011
Vela Spilja-Vela Luka	Rib	-19.2	9.0	3.2			Mesolithic	Croatia	Lightfoot et al. 2011
Grotta Addaura Caprara	Patella	-19.3	8.7	3.2	KIA-36053	8380 ± 40	Mesolithic	Sicily	Mannino et al. 2011
Grotta della Molara	Rib	-19.5	10.4	3.1	OxA-V-2364-39	9210 ± 40	Mesolithic	Sicily	Mannino et al. 2011
Grotta Addaura Caprara	Humerus	-19.6	9.7	3.4	KIA-36054	8330 ± 40	Mesolithic	Sicily	Mannino et al. 2011
Grotta della Molara	Metatarsal	-20.2	7.1	3.3	KIA-36052	8365 ± 40	Mesolithic	Sicily	Mannino et al. 2011
Grotta d'Oriente X	Ulna	-17.8	10.6	3.2	OxA-V-2364-37	8653 ± 39	Mesolithic	Sicily	Mannino et al. 2012
Grotta d'Oriente B	Rib	-18.8	11.2	3.3	KIA-36050	9395 ± 45	Mesolithic	Sicily	Mannino et al. 2012
Grotta d'Oriente B	Rib	-18.9	11.1	3.2	KIA-36049	9275 ± 45	Mesolithic	Sicily	Mannino et al. 2012
Grotta d'Oriente B	Rib	-19.0	11.6	3.2	KIA-36051	9440 ± 40	Mesolithic	Sicily	Mannino et al. 2012
Grotta dell'Uzzo	Cranium	-16.2	12.8	3.1			Mesolithic-Neolithic	Sicily	Mannino et al. 2015
Grotta dell'Uzzo	Humerus	-18.9	10.8	3.3			Mesolithic	Sicily	Mannino et al. 2015
Grotta dell'Uzzo	Rib	-19.0	11.6	3.3			Mesolithic	Sicily	Mannino et al. 2015

Grotta dell'Uzzo	Cranium	-19.1	8.7	3.3			Mesolithic	Sicily	Mannino et al. 2015
Grotta dell'Uzzo	Rib	-19.1	11.5	3.2			Mesolithic	Sicily	Mannino et al. 2015
Grotta dell'Uzzo	Rib	-19.1	11.7	3.5			Mesolithic	Sicily	Mannino et al. 2015
Grotta dell'Uzzo	Rib	-19.1	11.7	3.4			Mesolithic	Sicily	Mannino et al. 2015
Grotta dell'Uzzo	Rib	-19.3	8.7	3.1			Mesolithic	Sicily	Mannino et al. 2015
Grotta dell'Uzzo	Vertebra	-19.5	9.7	3.3			Mesolithic	Sicily	Mannino et al. 2015
Grotta dell'Uzzo	Rib	-19.5	9.8	3.3			Mesolithic	Sicily	Mannino et al. 2015
Grotta dell'Uzzo	Cranium	-19.7	9.7	3.3			Mesolithic	Sicily	Mannino et al. 2015
Grotta dell'Uzzo	Ulna	-19.7	10.8	3.3			Mesolithic	Sicily	Mannino et al. 2015
Grotta dell'Uzzo	Rib	-20.1	9.4	3.4			Mesolithic	Sicily	Mannino et al. 2015
Grotta dell'Uzzo	Rib	-20.3	9.9	3.6			Mesolithic	Sicily	Mannino et al. 2015
Grotta dell'Uzzo	Phalanx	-20.3	12.0	3.5			Mesolithic	Sicily	Mannino et al. 2015
Grotta dell'Uzzo	Rib	-20.5	9.4	3.5			Mesolithic	Sicily	Mannino et al. 2015
Grotta dell'Uzzo	Rib	-20.9	11.4	3.5			Mesolithic	Sicily	Mannino et al. 2015
Grotta dell'Uzzo	Rib	-21.2	11.3	3.5			Mesolithic	Sicily	Mannino et al. 2015
Pupićina Cave	Long bone fragment	-19.3	10.6	n.d.			Mesolithic	Croatia	Paine et al. 2009
Monte Leone		-18.0	9.9	n.d.			Mesolithic	Corsica	Pouydebat 1997

Cingle del Mas Nou	Neurocranium	-17.5	9.5	3.3	OxA-V-2360-29	6925 ± 35	Mesolithic	Spain	Salazar-García et al. 2014
Cingle del Mas Nou	Neurocranium	-17.8	9.2	3.3			Mesolithic	Spain	Salazar-García et al. 2014
Coves de Santa Maira	Scapula	-17.8	9.9	3.3	OxA-V-2360-26	8283 ± 37	Mesolithic	Spain	Salazar-García et al. 2014
Coves de Santa Maira	Humerus	-18.0	8.8	3.3			Mesolithic	Spain	Salazar-García et al. 2014
Coves de Santa Maira	Femur	-18.1	9.4	3.2			Mesolithic	Spain	Salazar-García et al. 2014
Penya del Comptador	Fibula	-18.2	7.6	3.2			Mesolithic	Spain	Salazar-García et al. 2014
Cingle del Mas Nou	Neurocranium	-18.3	9.8	3.2			Mesolithic	Spain	Salazar-García et al. 2014
Cingle del Mas Nou	Neurocranium	-18.4	7.9	3.2	OxA-V-2360-28	6897 ± 34	Mesolithic	Spain	Salazar-García et al. 2014
Cingle del Mas Nou	Neurocranium	-18.4	9.7	3.2			Mesolithic	Spain	Salazar-García et al. 2014
Cingle del Mas Nou	Neurocranium	-18.5	8.7	3.3			Mesolithic	Spain	Salazar-García et al. 2014
Cingle del Mas Nou	Neurocranium	-18.5	9.8	3.2			Mesolithic	Spain	Salazar-García et al. 2014
Cingle del Mas Nou	Neurocranium	-18.6	8.6	3.3			Mesolithic	Spain	Salazar-García et al. 2014
Cingle del Mas Nou	Neurocranium	-18.6	9.0	3.3			Mesolithic	Spain	Salazar-García et al. 2014
Penya del Comptador	Mandible	-18.6	7.6	3.2	OxA-V-2360-30	8829 ± 38	Mesolithic	Spain	Salazar-García et al. 2014
Penya del Comptador	Humerus	-18.7	7.8	3.3			Mesolithic	Spain	Salazar-García et al. 2014

Table 1: Published carbon and nitrogen stable isotope data and radiocarbon measurements obtained on Mesolithic human bone collagen throughout the Mediterranean basin. Note that only those data that had a C:N atomic ratio between 2.9-3.6 (DeNiro 1985) are listed. The exceptions are Araguina Sennola (Bocherens 1999), Pupićina Cave (Paine et al. 2009) and Monte Leone (Pouydebat 1997) in which no data were reported.
